# Quality of Life, Psychological Distress, and Nutritional Status of Polish Patients with Head and Neck Cancer Treated with Radiotherapy

**DOI:** 10.3390/jcm12020659

**Published:** 2023-01-13

**Authors:** Aleksandra Pytel, Anna Zielińska, Jakub Staś, Mariusz Chabowski

**Affiliations:** 1 Department of Nursing and Obstetrics, Division of Internal Medicine Nursing, Faculty of Health Science, Wroclaw Medical University, 5 Bartla Street, 51-618 Wroclaw, Poland; 2 Lower Silesian Oncology Center, 12 Hirszfeld Square, 53-413 Wroclaw, Poland; 3 Student Research Club No 180, Faculty of Medicine, Wroclaw Medical University, 50-367 Wroclaw, Poland; 4 Department of Nursing and Obstetrics, Division of Anaesthesiological and Surgical Nursing, Faculty of Health Science, Wroclaw Medical University, 5 Bartla Street, 51-618 Wroclaw, Poland; 5 Department of Surgery, 4th Military Teaching Hospital, 50-981 Wroclaw, Poland

**Keywords:** quality of life, nutrition, head and neck cancer, radiotherapy

## Abstract

Introduction. Head and neck cancer (HNC) is a global epidemiological and clinical problem. In 2020, it was the seventh most common type of cancer worldwide. In 2019, HNC was the fourth most common cause of cancer death among men in Poland. Radiotherapy plays an important role in the treatment of patients with HNC at all clinical stages. However, it is associated with a significant rate of early and late adverse effects. As head and neck cancers are located close to vital anatomical structures, both the local progression of the disease and the treatments used can cause serious problems for patients with HNC, reducing their health-related quality of life (HRQoL) as well as increasing the risk of depressive disorders. Despite this, the current literature lacks research on these aspects of the therapeutic process in the Polish population. The aim of this study was to assess the early impact of radiotherapy on HRQoL outcomes, psychological distress, nutritional status, and overall performance of patients with HNC. Methods. The study was carried out among 85 patients with HNC treated in the Inpatient Radiotherapy Unit of the Radiotherapy Department of the Lower Silesian Oncology Center in Wrocław. The patients were asked to complete a set of questionnaires, including the EORTC QLQ-H&N35, the BDI, the NRS-2002, and the ECOG scale, at two time points: before the initiation of radiotherapy treatment and after a course of radiotherapy. The period between the assessments was 7 to 8 weeks. Results. Our findings demonstrated a negative impact of radiotherapy on scores in all the cancer-specific symptom and functioning scales used in the study. As regards functioning scales, the largest differences were observed for senses and swallowing, whereas with symptoms scales, the largest differences were noted for “sticky saliva” and “dry mouth”. Over half of the patients included in the study required nutritional support after radiotherapy treatment. We found statistically significant differences in the levels of depressive symptom severity before and after radiotherapy treatment. Conclusion. The present study showed significant changes in the physical and psychological functioning and nutritional status of the patients with HNC studied after radiotherapy treatment, which may have an impact on the effectiveness of cancer treatment.

## 1. Introduction

Head and neck cancer (HNC) is a global epidemiological and clinical problem. In 2020, it was the seventh most common type of cancer worldwide [1]. The incidence of HNC is noticeably higher in regions with a low level of economic development, where HNC is the most common type of cancer [2]. In Central and Eastern European countries, there are also significant adverse epidemiological trends in HNC [3]. In 2019, HNC was the fourth most common cause of cancer death among men in Poland [4].

HNCs include malignancies located within the lips, tongue, oral cavity, nasal sinuses, pharynx, salivary glands, and larynx. Despite the diverse location, head and neck cancers are a relatively homogeneous group in terms of histopathological classification. In over 90% of cases, HNCs are squamous cell carcinomas. Typical risk factors for the development of squamous cell carcinomas of the head and neck (HNSCC) include tobacco smoking and excessive alcohol consumption. In recent years, the relationship of HPV infection, in particular the HPV-16 serotype, with an increased risk of HNC has been proven. HPV-related HNCs are clinically and epidemiologically distinct from cancers associated with classical risk factors. HPV-related cancers tend to occur at a younger age in patients with no history of tobacco or alcohol exposure. They are also characterised by a milder course and less frequent distant metastases [5].

The management of patients with HNC is multidisciplinary in nature. Radiotherapy is a viable treatment option for patients with HNC at all clinical stages, both alone (cT1-2 cN0 cM0) and in combination with surgery and chemotherapy [6]. Despite the continued advances in personalisation of therapy and good treatment outcomes, radiotherapy treatment is still associated with a significant rate of early and late adverse effects [7,8].

As head and neck cancers are located close to vital anatomical structures, both the local progression of the disease and the treatments used can cause serious problems for patients. HNCs and their treatment have, especially in advanced cases, an extremely negative impact on the quality of life [9]. The impact of the disease on functioning, as well as aesthetic issues, contributes to difficulties in adapting the patient to the state after radical treatment. Patients may experience severe acute and late radiation reactions causing dysfunction of the head and neck organs. Their typical example is drying of the mucous membranes (xerostomia), which is the result of damage to the secretory function of the salivary glands. Impaired salivation causes discomfort and also makes it difficult to eat, leading to malnutrition [10]. It can also cause dental problems and the need for specialist treatment [11]. Patients with HNC may also experience dysphagia related to the complex damage to the structure and function of the aerodigestive tract [12,13].

Difficulties eating, speaking, and breathing; hearing problems; and changes in the perception of one’s body image affect all areas of personal and professional life [14,15,16]. Therefore, a thorough multifactor analysis of the patient’s quality of life and their ability to independently function should be carried out before, during, and after treatment for HNC [17].

The aim of this study was to assess the early impact of radiotherapy on health-related quality of life (HRQoL) outcomes, psychological distress, nutritional status, and overall performance of patients with HNC.

## 2. Materials and Methods

### 2.1. Study Population

The study was carried out among 85 patients treated in the Inpatient Radiotherapy Unit of the Radiotherapy Department of the Lower Silesian Oncology Center in Wrocław. The criteria for inclusion in the study were as follows: age 18 years or over, diagnosis of primary HNC, radical treatment with radiotherapy, chemoradiotherapy or adjuvant radiotherapy after surgical resection, and provision of voluntary consent to participate in the study. The study was approved by the Bioethics Committee of the Wrocław Medical University (approval no. KB—632/2018).

The study was carried out between July 2018 and February 2019. All participants provided informed written consent to participate in the study. The participants were asked to complete a set of questionnaires twice: before the initiation of radiotherapy treatment and after a course of radiotherapy. The period between the assessments was between 7 and 8 weeks.

### 2.2. European Organization for Research and Treatment of Cancer Quality of Life Questionnaire for Head and Neck Cancer 35 (EORTC QLQ-H&N35)

The quality of life of the patients studied was assessed using the Polish language version of the European Organization for Research and Treatment of Cancer Quality of Life Questionnaire for Head and Neck Cancer 35 (EORTC QLQ-H&N35). The questionnaire is used to assess the overall quality of life of patients with HNC as well as the symptoms specific to HNC and those related to its treatment [18,19].

The EORTC QLQ-H&N35 includes 35 questions, 30 of which assess head and neck pain, problems with swallowing, senses problems, the presence of coughing and hoarseness, problems with feeling ill, trouble with social eating, trouble with social contact, and sexuality during the past week. Patients respond to those questions on a 4-point scale. The remaining 5 questions concern the use of painkillers and nutritional supplements, the use of a feeding tube, weight loss, and weight gain. Those questions request a yes or no answer.

Patient responses are summed for physical, functional, emotional, and social features. They enable the assessment of the impact of cancer and its treatment on particular areas of the patient’s functioning.

The higher the total score, the greater the severity of problems relating to the illness and its treatment and, thus, the poorer the quality of life. A difference (∆) in score of 10 points or more on a scale from 0 to 100 between the two assessment time points (before and after radiotherapy treatment) was considered to be clinically significant, indicating deterioration or improvement in quality of life. This cut-off value is commonly used in studies. A difference (∆) of 20 points or more is considered to be particularly significant [20].

### 2.3. Beck Depression Inventory (BDI)

The severity of depressive symptoms was assessed using the Beck Depression Inventory (BDI). The BDI comprises 21 groups of statements concerning mental status and somatic depressive symptoms. Patients are asked to choose one statement in each group the best describes the way they have been feeling during the past week. Each answer is scored on a scale of 0–3. The higher the score, the higher the severity of depressive symptoms. The final score is a sum of all item scores and ranges between 0 and 63 points. Scores below 12 indicate no depression, 12–19 indicate mild depression, 20–25 indicate moderate depression, and 26–63 indicate severe depression [21]. The BDI is commonly used as a screening tool for a depressive disorder in cancer patients, the accuracy of which has also been confirmed among patients with HNC [22,23].

### 2.4. Nutrition Risk Screening 2002 (NRS-2002) and Body Mass Index (BMI)

The risk of malnutrition was assessed using the Nutrition Risk Screening 2002 (NRS-2002). The NRS-2002 is a tool routinely used to assess nutritional status. It is recommended by the European Society for Clinical Nutrition and Metabolism (ESPEN). The NRS-2002 assesses nutritional status (weight loss and coverage of energy needs) and the potential increase in energy needs resulting from an increase in disease severity. Each of the two components is assigned a score of 0–3. For patients aged over 70 years, an additional 1 point is added to the total score. A score ≥3 indicates the need for nutritional support [24].

In order to assess the nutritional status of the patients studied, anthropometric measurements, including height and weight, were also taken. They were then used to calculate BMI, i.e., the ratio of body weight in kilograms to height in metres squared. The assessment was carried out twice (before and after radiotherapy treatment).

### 2.5. ECOG Performance Status Scale

The overall performance status of the patients studied was assessed using the Eastern Cooperative Oncology Group (ECOG) scale. The scale is used to assess the overall condition of cancer patients. It ranges from grade 0 (fully active) to 5 (dead) [25].

Our own questionnaire used in the study included questions concerning sociodemographic data, such as age, sex, education, and place of residence.

### 2.6. Statistical Analysis

Our statistical analysis involved calculating scores on particular dimensions of the standardised scales used in the study. Descriptive statistics, i.e., means, minimum and maximum values, medians and standard deviations, were calculated. The *t*-test or Wilcoxon test was used to compare scores (quantitative variables) of the pre- and post-treatment assessments. The significance level was set at *p* < 0.05. The analyses were performed using Statistica 13.0 (TIBCO, Palo Alto, Santa Clara, CA, USA).

## 3. Results

### 3.1. Sociodemographic Characteristics of the Patients Studied

The study included 85 patients with HNC (55 men and 30 women) treated with radiotherapy, of whom the largest proportion were aged 56–65 years (38.8%). The vast majority of the patients had secondary education (41.2%) and lived in cities of up to 20,000 inhabitants (42.3%) (Table 1).

### 3.2. Analysis of the Results of the Assessment of Overall Condition and Performance Status Using the ECOG Scale before and after Radiotherapy Treatment

Table 2 shows a comparison of the ECOG scores between the first and second measurements; a statistically significant increase in the ECOG scores was observed (*p* < 0.001).

The analysis of the overall condition and performance status of the patients studied using the ECOG scale showed a negative impact of radiotherapy treatment on the performance status of the patients. A decrease in the score on the scale was observed for 2 patients (negative ranks), whereas an increase in the score was observed for 57 patients (positive ranks). The higher the score on the scale, the worse the performance status. The differences were statistically significant (*p* < 0.05). The number of ties was 26 (Table 3).

### 3.3. Results of the Assessment of Quality of Life Using the EORTC QLQ-H&N35

Table 4 shows the results of the *t*-test for dependent samples. The table shows the mean values of the variables tested, including their statistical significance and standard deviation. An analysis of mean values made it possible to determine differences in the compared variables between the two time points (before and after radiotherapy treatment). A difference (∆) of 10 points or more was considered to be clinically significant, indicating deterioration in the quality-of-life parameter analysed. A difference of 20 points or more, according to the above recommendations, is considered to be particularly significant.

The baseline assessment of the QoL in terms of the scales of the EORTC QLQ–H&N35 questionnaire showed that the patients had the highest severity of symptoms in the following domains: teeth 50.0 ± 0.0; weight gain 48.5 ± 5.9; feeding tube 46.8 ± 8.4; and pain 28.3 ± 25.2. They had the lowest-intensity symptoms in the domains: dry mouth 25.0 ± 0.0; senses 26.3 ± 4.7; and trouble with social contact 28.5 ± 6.5.

The post-treatment reassessment showed the highest severity of symptoms in the following domains: sticky saliva 76.2 ± 23.4, dry mouth 75.9 ± 25.1, and senses 71.2 ± 24.6; and the lowest intensity in the domains of painkillers 25.9 ± 4.6, nutritional supplements 28.2 ± 8.4, and weight loss 29.7 ± 9.8.

The greatest differences in pre- and post-treatment measurements were noted for the domains of sticky saliva (Δ = 51.2), dry mouth (Δ = 50.9), and senses (Δ = 44.8).

### 3.4. Analysis of Changes in BMI between the Two Assessment Time Points (before and after Radiotherapy Treatment)

Table 5 shows the comparison of the BMI between the first and second measurements; a statistically significant decrease in the BMI scores was observed (*p* < 0.001).

A decrease in BMI was observed for 79 patients (negative ranks), whereas an increase in BMI was observed for 5 patients (positive ranks). There was one tie. The median BMI at the first assessment time point was 22.70, whereas the median BMI at the second assessment time point was 20.90. The differences observed were statistically significant (*p* < 0.05) (Table 6).

### 3.5. Analysis of Nutritional Risk

An analysis of whether the patients studied required nutritional support was carried out using the NRS-2002 at two time points: before and after radiotherapy treatment. The differences observed were statistically significant (*p* < 0.05). The baseline assessment of nutritional status in the NRS 2002 scale showed the need for nutritional intervention in only 9.4% of patients. Reassessment after radiotherapy showed the need for nutritional intervention in 55.3% of the study group (Table 7).

### 3.6. Assessment of the Severity of Depressive Symptoms

Statistically significant differences in the severity of depression were observed between the two assessment time points (*p* < 0.05) (Table 8).

A decrease in the severity of depressive symptoms was observed for 13 patients (negative ranks), whereas an increase in the severity of depressive symptoms was observed for 71 patients (positive ranks). There was one tie. The median BDI score at the first assessment time point was 5.00, whereas the median BDI score at the second assessment time point was 11.00. The differences observed were statistically significant (*p* < 0.05) (Table 9). After completion of radiotherapy treatment, mild depression was identified in 31 patients (36.5%) and moderate depression in 8 patients (9.4%).

## 4. Discussion

The issue of the negative impact of HNC and its treatment on the quality of life, depressive symptoms, and nutritional status of patients is complex and important from the point of view of both public health and clinical practice. The changing epidemiological trends in HNC, and in particular the rising incidence of HPV-related HNC, are reflected in the increase in the number of HNC cases among people aged between 40 and 59 years, and notably in men [26,27,28]. However, the public awareness of HNC, both in the USA and in central and eastern European countries, such as Poland, is relatively low [29,30]. Studies have shown that the productivity loss associated with HNC is higher than that associated with other types of cancer [31,32]. Moreover, the treatment of HNC can produce a higher financial burden, both to healthcare systems and to patients, compared with other cancers [33,34]. This underscores the need for careful analysis of all variables associated with the quality of life and functioning of patients with HNC.

Pretreatment quality of life is a significant prognostic factor for survival in patients with HNC [35]. In addition, HRQoL is, alongside mortality, survival, and recurrence rates, an important measure of treatment outcomes [36]. Despite this, there is currently a very limited number of publications in this field concerning the Polish population in the literature.

Our study showed a significant decrease in the immediate QoL outcomes of patients with HNC treated with radiotherapy. Our findings demonstrated a negative impact of acute radiotherapy toxicity on scores in all the cancer-specific functioning scales used in the study. The largest differences were found for the functioning scales relating to senses and swallowing. In addition, a significant deterioration in scores was found for particular cancer-related symptom scales. The largest differences were found for the “sticky saliva” and “dry mouth” scales.

Milecki et al. assessed the changes in the HRQoL in patients with HNC treated with radiotherapy at 12 months after completion of treatment. In terms of the QLQ-H&N35 questionnaire, the authors reported a statistically significant deterioration in the QoL for scales of “dry mouth”, “weight loss”, “senses” “sticky saliva”, “opening mouth”, and “painkillers”. The effect of tumour location on QoL deterioration was statistically significant on the scales “speech” (higher deterioration in patients whose tumour was located in the larynx and hypopharynx) and “opening mouth” (higher deterioration when tumour was located in the oral cavity) [37].

A comparison of the results of our own research with the those of Milecki et al. points to a greater severity of swallowing difficulties and troubles with social eating among patients immediately after the completion of treatment with radiotherapy.

Problems with chewing and swallowing necessitate a switch to a more liquid diet as well as the use of nutritional supplements, which, in some cases, may necessitate the use of a feeding tube. The eating difficulties experienced by patients with HNC are associated not only with a compromised nutritional status, but also with the feelings of shame when eating in front of others [38]. In the present study, only two patients reported that they often or very often had problems with social eating prior to the treatment, whereas such problems were reported by more than one-third of patients after radiotherapy treatment.

Dry mouth and sticky saliva are among the most common complaints of patients with HNC treated with radiotherapy, which was also confirmed by the present study. Radiotherapy of the head and neck may cause damage to salivary glands and result in a reduction in saliva production, which has an impact not only on the comfort of patients but also on their chewing ability and taste sensation [38]. Dysgeusia and ageusia cause patients to quickly lose interest in food, which results in compromised nutritional status and contributes to weight loss [39].

Over half of the patients included in the present study required nutritional support after radiotherapy treatment. Post-treatment weight loss was reported in 79 of the 85 patients studied. The nutritional status of patients with HNC has a significant impact on their prognosis [40]. Malnutrition and progressive cachexia are common problems in cancer patients [41]. Patients with HNC are at particular risk of malnutrition due to the location of the cancer and local-treatment-related toxicity [42]. Nutritional care should be an integral part of the management of patients with HNC, both during and after treatment [43]. Intensive nutritional care makes it possible to effectively prevent deterioration in the quality of life of patients with HNC resulting from radiotherapy treatment [44,45,46,47].

Participants in this study were assessed for the presence of depressive symptoms using the BDI questionnaire. To the best of our knowledge, this is the first study assessing the severity of depressive symptoms among patients with HNC in the Polish population. A mild-to-moderate depressive episode was identified in 45,9% of patients.

The severity of depressive symptoms may also have an impact on the nutritional status of patients with HNC. However, in HNC, depressive symptoms and nutritional status influence each other in a dynamic, rather than static, interplay over time [48]. The results of the present study showed a statistically significant increase in the severity of depressive symptoms in the patients studied, as measured immediately after radiotherapy treatment. Longitudinal studies of depressive symptoms in patients with HNC undergoing radiotherapy showed that the severity of depressive symptoms in those patients is highest during the treatment [49]. The results of the present study showed a mild or moderate level of depressive symptom severity, as assessed using the BDI immediately after radiotherapy treatment, in 52.7% of men and 33.3% of women included in the study. The association between sex and the severity of depressive symptoms was not statistically significant. Other studies confirmed a significant prevalence of depressive disorders among patients with HNC [22].

The side effects of radiotherapy and the resulting reduction in quality of life and increase in the severity of depressive symptoms may cause the patient to no longer want to continue treatment, which may lead to disease progression or recurrence [50]. Numerous studies have demonstrated a negative impact of interruptions in radiotherapy for HNC on the local control of cancer, survival, and length of survival without recurrence [51,52,53].

The awareness of the negative consequences of radiotherapy treatment and their impact on the patient’s quality of life allows appropriate preventive measures to be taken more quickly, thus increasing the patient’s motivation to complete treatment and their chances of making a full recovery. Complete elimination of the side effects of radiation therapy may not be possible for most patients. In recent years, significant progress has been made in alleviating the symptoms specific to HNC treated with radiotherapy. Despite this, the consequences of radiotherapy, such as xerostomia and salivary hypofunction, are still a significant problem. Reducing radiation damage to the salivary glands, for example, by using intensity-modulated radiation therapy (IMRT), is the primary way to reduce the occurrence of dry mouth [54]. The use of stem cells to restore salivary gland function remains a promising direction [55]. Both early and late speech-language pathologist interventions appear to be beneficial in reducing the severity of dysphagia among patients with HNC treated with radiotherapy [56]. Regarding the nutritional status of patients with HNC, dietary counselling may play a role in counteracting the negative impact of treatment [57]. A study by Samuel et al. showed a beneficial effect of rehabilitation with exercise on the functioning and quality of life of patients with HNC treated with radiochemotherapy [58].

The study has several limitations. One is the lack of data definition and analysis based on data on tumour type, stage, location, etc. Future studies should include these data in their analyses. Another limitation of the study is the time of assessment immediately after the end of radiotherapy, which allows for the assessment of the impact of very early toxicity of treatment on QoL, but does not allow for examining the peak of side effects related to progressive fibrosis and scarring. In addition, it would be worth designing a study with a control group in the future. The limitations of this study result from the relatively small study group and the high clinical heterogeneity of patients (e.g., treatment with radiotherapy alone, chemoradiotherapy, or radiotherapy as an adjunct to surgery). Additional studies taking into account the impact of sociodemographic factors and clinical characteristics are required to further specify the risk factors limiting the QoL of patients with HNC.

## 5. Conclusions

The present study found significant changes in the physical and psychological functioning and nutritional status of the patients with HNC studied immediately after radiotherapy treatment. The early adverse effects of treatment experienced by patients may reduce the effectiveness of cancer treatment. Malnutrition, which may be a consequence of disorders that both impair eating and increase in the severity of depressive symptoms, especially anorexia nervosa, increases the risk of treatment complications and cancer-related death, whereas a deteriorating quality of life and an increase in the severity of depressive symptoms may discourage patients from continuing treatment, resulting in disease progression or recurrence. The results suggest potential targets for future interventions to improve outcomes of patients with HNC through actions aimed at improving the HRQoL and nutritional status.

## Figures and Tables

**Table 1:** General sociodemographic characteristics of the patients.

Feature (Variable)	*n* (%)
1. Sex	
Female	30 (35.3%)
Male	55 (64.7%)
2. Age (years)	
18–35	2 (2.4%)
36–45	12 (14.1%)
46–55	12 (14.1%)
56–65	33 (38.8%)
Over 65	30 (30.6%)
3. Education	
Primary	2 (2.3%)
Vocational	34 (40%)
Secondary	35 (41.2%)
Tertiary	14 (16.5%)
4. Place of residence	
Rural area	12 (14.1%)
City of up to 20,000 inhabitants	36 (42.3%)
City of between 21,000 and 50,000 inhabitants	27 (31.8%)
City of more than 50,000 inhabitants	10 (11.8%)

**Table 2:** Assessment of performance status using the ECOG scale.

	Descriptive Statistics		
Performance Status	Me	Min.	Max.
ECOG scale—1st assessment	0.00	0.00	2.00
ECOG scale—2nd assessment	1.00	0.00	4.00
*p*-value		<0.001	

Me—median; Min.—minimum score; Max.—maximum score; *p*—statistical significance (Wilcoxon signed-rank test).

**Table 3:** Ranks of performance status (ECOG).

		N
ECOG scale—2nd assessment—ECOG scale—1st assessment	Negative ranks	2
	Positive ranks	57
	Ties	26
	Total	85

**Table 4:** Analysis of the determinants of quality of life (EORTC QLQ–H&N35).

Variable		Mean	Standard Deviation	Difference of Means (Δ)	*p*-Value *
p1	Swallowing (before RT)	31.4	12.0	35.0	<0.001
	Swallowing (after RT)	66.4	21.8		
p2	Senses (before RT)	26.3	4.7	44.8	<0.001
	Senses (after RT)	71.2	24.6		
p3	Speech (before RT)	31.9	10.3	25.0	<0.001
	Speech (after RT)	56.9	21.0		
p4	Trouble with social eating (before RT)	30.9	12.1	36.0	<0.001
	Trouble with social eating (after RT)	66.9	21.8		
p5	Trouble with social contact (before RT)	28.5	6.8	17.3	<0.001
	Trouble with social contact (after RT)	45.8	15.4		
p6	Sexuality (before RT)	34.0	16.0	11.5	<0.001
	Sexuality (after RT)	45.5	20.0		
p7	Teeth (before RT)	50.0	0.0	0.9	
	Teeth (after RT)	50.9	23.3		
p8	Opening mouth (before RT)	50.0	0.0	16.5	
	Opening mouth (after RT)	66.5	25.5		
p9	Dry mouth (before RT)		0.0	50.9	
	Dry mouth (after RT)	75.9	25.1		
p10	Sticky saliva (before RT)	25.0	0.0	51.2	
	Sticky saliva (after RT)	76.2	23.4		
p11	Coughing (before RT)	33.8	16.2	30.3	<0.001
	Coughing (after RT)	64.1	24.2		
p12	Felt ill (before RT)	33.2	11.8	26.2	<0.001
	Felt ill (after RT)	59.4	20.8		
p13	Painkillers (before RT)	43.8	11.5	17.9	<0.001
	Painkillers (after RT)	25.9	4.6		
p14	Nutritional supplements (before RT)	44.1	10.7	15.9	<0.001
	Nutritional supplements (after RT)	28.2	8.4		
p15	Feeding tube (before RT)	46.8	8.4	0.0	>0.05
	Feeding tube (after RT)	46.8	8.4		
p16	Weight loss (before RT)	38.8	12.5	9.1	<0.001
	Weight loss (after RT)	29.7	9.8		
p17	Weight gain (before RT)	48.5	5.9	0.0	>0.05
	Weight gain (after RT)	48.5	5.9		

* differences were analysed using the *t*-test for dependent samples.

**Table 5:** BMI assessment.

	Descriptive Statistics		
BMI	Me	Min.	Max.
BMI—1st assessment	22.70	16.20	33.40
BMI—2nd assessment	20.90	15.20	32.70
*p*-value		<0.001	

Me—median; Min.—minimum score; Max.—maximum score; *p*—statistical significance (Wilcoxon signed-rank test).

**Table 6:** Ranks of BMI assessment.

		N
BMI—2nd assessment—BMI—1st assessment	Negative ranks	79
	Positive ranks	5
	Ties	1
	Total	85

**Table 7:** Assessment of nutritional risk before and after radiotherapy treatment.

Assessment of Nutritional Risk (1st Assessment)	Frequency	Percentage
Nutritional support is indicated	8	9.40%
Consider conservative management, repeat the test in 1 week	77	90.60%
Assessment of nutritional risk (2nd assessment)	Frequency	Percentage
Nutritional support is indicated	47	55.30%
Consider conservative management, repeat the test in 1 week	38	44.70%

**Table 8:** Beck Depression Inventory.

	Descriptive Statistics		
Beck Depression Inventory	Me	Min.	Max.
BDI—1st assessment	5.00	0.00	35.00
BDI—2nd assessment	11.00	0.00	39.00
*p*-value		<0.001	

Me—median; Min.—minimum score; Max.—maximum score; *p*—statistical significance (Wilcoxon signed-rank test).

**Table 9:** Ranks of BDI.

		N
BDI—2nd assessment—BDI—1st assessment	Negative ranks	13
	Positive ranks	71
	Ties	1
	Total	85

## Data Availability

The data are available from the authors of the manuscript after contacting the corresponding author.
